# Dental Trade Association

**Published:** 1889-08

**Authors:** R. T. Morrison, Lee S. Smith


### DENTAL TRADE ASSOCIATION.

### An Explanation.

At the last annual meeting of the American Dental Trade Association it was ordered that the following statement be published.

Following the organization of the American Dental Trade Association, seven years ago, there was for a time an apprehension among dentists that its effect, if not its object, would be to increase the prices of dental goods and in various ways to oppress the dental profession. This misconception of its purpose was of course diligently encouraged by those who preferred not to join the association. As time passed, however, the great majority of the profession came to recognize that the practical working of the association, so far from being in any way oppressive to them, was really an advantage. A very few have chosen to maintain the attitude of martyrs, and are still endeavoring to prejudice the minds of dentists regarding the association, its objects, and its results.

Within the last year or two there has been more than usual effort to disseminate views in opposition to the association. Not only in dental society meetings and in dental journals, but in newspapers there have been statements so at variance with the facts as to have all the effect of deliberate misrepresentation. The terms "dental trust," "pool," "combination," etc., have been employed with the view of arousing antipathy and antagonism, through the impression sought to be created that the association was open to the criticisms merited and bestowed on other organizations whose objects were conceded to be dishonorable if not unlawful. The association has been referred to as "a combination to prevent competition, to perpetuate monopoly, and to squeeze the dentists."

In a published report of a union dental meeting held in Boston in July, 1888, the following preamble and resolutions are reported as having been adopted:

"*Whereas*, certain manufacturers and dealers in dental instruments and materials have formed a combination known to the profession as the Dental Trade Association; and

"*Whereas*, the forming of such combination can only be an obstacle, retarding progress in the direction of scientific investigation, improvement and higher professional attainment; therefore

"*Resolved*, that we consider the forming of such combination to be a reflection on the scientific and professional character of our profession.

"*Resolved*, that we invite all members of said combination to withdraw from the same, and we pledge them our hearty interest and support."

It is not easy to understand how the formation or the continuance of an association of merchants and manufacturers can retard "scientific investigation," interfer with "higher professional attainments," or "be a reflection on the scientific and professional character" of a profession. The resolutions were evidently prepared by one whose zeal was not tempered by knowledge, in an attempt to attack those who had no opportunity of defense or reply, and were adopted, without consideration, by those who fancied they had a grievance, without the ability to state its precise character. Most of the attacks that have been made upon the association have been similarly vague and meaningless, and similarly calculated to impress the unprejudiced that any lack of a true "scientific and professional character" in their authors had other explanation than the existence of a trade association, and must have antedated its organization.

The association at this, its first annual meeting since the publication of the quoted resolutions, and other like attacks, deems it fitting to present in reply a brief account of its organization and objects.

The American Dental Trade Association was organized between seven and eight years ago. Its germ originated during a dental convention at a meeting, without pre-concert, of five dealers, who were at that time in the habit of canvassing to some extent the same territory. The business in that section had been for some years in a most unsatisfactory condition. The original idea was limited to the suggestion of a local organization or board of trade, for the purpose of arriving at a better understanding among themselves. The subject, however, broadened, and it was finally decided to invite all dental dealers and manufacturers to unite in a trade association. After considerable discussion a plan of organization was matured, and at a meeting held at Niagara Falls in June, 1882, the association was formed. It now numbers about seventy-five members, including most of the leading manufacturers and dealers in the trade.

Previous to this there had been very little harmony or kindly feeling among those engaged in the business. Although in active competition, most of them were strangers to each other; there were jealousies and suspicions which led to doubtful dealing. There was likewise a time when there were no dental societies; when each man was working solely for himself, perhaps jealous of his brother practitioner, and unwilling to aid him professionally. All dentists now know the value of association, of a code of ethics, and of occasional meetings for discussions and friendly intercourse.

In almost every branch of business there are associations and boards of trade for the establishment of commercial ethics applicable to their specialty, and for the cultivation of kindly feeling. The general sentiment of the community is that such associations are desirable and that their effect is beneficial. It remains for a few dentists to deny to those with whom they have their principal dealings that which they highly value for themselves, and which is freely accorded by the whole community to other branches of mercantile business.

The objects of the Dental Trade Association are set forth in the second article of its constitution, as follows:

"The objects of this association are to reform abuses; to secure unity of action; to promote a friendly intercourse between its members; to avoid and adjust, as far as practicable, differences and misunderstandings between them, and, generally, to advance the interests of the trade in dental goods in the United States."

Allusion has been made above to the inharmony which existed, and to some of the abuses which obtained between the members before their association, and which it was desirable to remove. The chief abuse which had grown up in the dealings of the members with the dental profession was discrimination of the rankest kind. It required the authority of the United States government to put a stop to unjust discriminations of the transportation companies of the country, and the interstate law has been everywhere approved as a long step in the right direction. The American Dental Trade Association undertook, of its own motion, to put a stop to discriminations in their own line of business, and to place all who dealt with its members upon the same plane of honorable and equitable treatment. Before that time there were, as there are now, regular list prices for goods, and the theory was that there were no discounts allowed from these prices, except in the case of a few articles which were charged at a lower rate when certain quantities were bought at one time. In practice, this theory of no discounts was enforced at least nine times out of ten. The exceptions were when two or more eager salesmen were bidding against each other for the sale of a chair, an outfit, or large lot of goods, when oftentimes prices were accepted that left the dealer little or no profit. One case is on record where three dealers were bidding against each other for the sale of a chair, and the unfortunate one who finally made the sale sold the chair at precisely what he paid for it, and lost the freight from the factory to his store in the West. The correct theory of honorable mercantile dealing is that both parties shall be benefited by it, and although every one likes to buy as cheaply as possible, it is not believed that any fair-minded dentist would feel comfortable in the belief that circumstances were compelling his dealer to serve him without profit, any more than he would feel satisfied to accept a fee from a patient without having rendered him beneficial service.

The unjust discrimination is apparent from the fact that the very next customer for a chair who presented himself would have been charged full list price without any discount whatever, if he went to the dealer confidingly, and other dealers could be kept from knowing his want. It was the large majority of confiding buyers, who simply sent in their orders without question, who were discriminated against, while comparatively few were favored with discounts. The Association corrected this by establishing list prices for time sales, and allowing what had never been generally done before, a discount for prompt cash of five per cent. on bills of $50 and over, and ten per cent. on bills of $150 on all goods except precious metals and such as were sold at a rebate for quantity, as teeth in lots. These discounts are now allowed to every one who pays cash. The result is that the dentists, as a body, pay less for their supplies, on the same list prices, than they did under the former system of discriminations in favor of a few, and the business is placed on an honorable, dignified basis.

Neither at the formation of the Association, nor at any time since, has the price of a single article been advanced because of its operations. On the contrary, a comparison of the rates of today with those prevailing eight years ago will show many, and in some instances large, reductions. Further, these reductions have almost all been brought about by competition inside the Association. All the talk about "combination," "dental trust," "pool," etc., as affecting prices, is absolulely baseless. There is but one rule of the Association that bears at all upon prices. Manufacturers, who are in almost every instance retailers as well, fix the retail prices of their own goods, as they always did, but the members who handle those goods agree to abide by the manufacturers' prices, which formerly they did not always do. In other words, the manufacturers permit the dealers all over the country to make a profit on the sale of their productions, at their prices, on the simple agreement that they shall not be undersold on their own goods. But the manufacturer can change his prices whenever he chooses, without let or hindrance, the only stipulation being that he will notify those who are dealing in his goods, that they may conform to the changes. Furthermore, in the case of two or more manufacturers making the same grade of goods, each is free to make his own rates without any reference to the others. Thus the White Company and Mr. Justi have at present the same list of prices for teeth, but each house can change its rates at will, without consultation with the other, or with any one in the Association.

Competition is also unrestricted; there is in no sense any pooling of interests, any limitation of production. Each member of the Association pushes his business "for all there is in it," just as though no Association was ever thought of. It could not be held together for a week on any other basis. It has been held together all these years on this foundation by a better acquaintance with each other than formerly, by mutual respect and friendly intercourse, by honorable dealings with each other, and by absolute non-discrimination.

Can any fair-minded man, after the above candid statement of facts, find any ground for condemning the Association? Must it not be admitted that it has placed the business on a better foundation and on a more honorable plane than it ever before occupied? That it has operated and will continue to operate to the benefit of the dental profession is our profound conviction.

It must be conceded that it was a benefit to the profession to do away with discriminations; and the Association has been of positive advantage to the very large number of dentists that are remote from the manufacturers and larger dealers, by encouraging their local dealers and enabling them to carry a larger stock to meet current, every-day wants.

It would be a step backward to return to the condition of the trade that existed in certain sections of the country eight to ten years ago. If the Association should be broken up it would not eventually benefit the profession, and it would not be the larger dealers and manufacturers who would chiefly suffer by it. Instead of being close monopolists, as has so often been charged, the larger manufacturers have to a certain extent allowed their hands to be tied by the Association, for the benefit of the general trade, and particularly of the smaller local dealers all over the country. Who does not realize that in any general scramble for business, regardless of whoever stood in the way, the greater capital and facilities of the large houses would speedily overcome the smaller ones, and thus give to the former, in the end, a much greater opportunity of "monopolizing" than is possible under the present system? With this statement of its objects and methods the American Dental Trade Association confidently appeals to the good sense of the dentists of the country for an unprejudiced acceptance of its well-meant efforts to help and not hinder the advancement of dental science and art, while seeking to establish and maintain a code of business ethics, as essential in its field as is the professional code to professional men.

THE AMERICAN DENTAL TRADE ASSOCIATION,
R. T. MORRISON, President.
NEW YORK, June 20, 1889. LEE S. SMITH, Secretary.

